# Assessment of genetic variability in captive capuchin monkeys (Primates: Cebidae)

**DOI:** 10.1038/s41598-021-86734-w

**Published:** 2021-03-31

**Authors:** Mariela Nieves, María Isabel Remis, Carla Sesarini, Diana Lucrecia Hassel, Carina Francisca Argüelles, Marta Dolores Mudry

**Affiliations:** 1 Grupo de Investigación en Biología Evolutiva (GIBE), Departamento de Ecología, Genética y Evolución (DEGE), Facultad de Ciencias Exactas y Naturales, Universidad de Buenos Aires, Intendente Güiraldes 2160, C.A.B.A., C1428EGA Buenos Aires, Argentina; 2 Instituto de Ecología, Genética y Evolución de Buenos Aires (IEGEBA), CONICET-Universidad de Buenos Aires, Intendente Güiraldes 2160, C.A.B.A., C1428EGA Buenos Aires, Argentina; 3 Genética de la Estructura Poblacional (GEP), Departamento de Ecología, Genética y Evolución (DEGE), Facultad de Ciencias Exactas y Naturales, Universidad de Buenos Aires, Intendente Güiraldes 2160, C.A.B.A., C1428EGA Buenos Aires, Argentina; 4 Grupo de Investigación en Genética Aplicada (GIGA), Departamento de Genética, Facultad de Ciencias Exactas, Químicas y Naturales. Instituto de Biología Subtropical (IBS)- Nodo Posadas, Consejo Nacional de Investigaciones Científicas y Técnicas (CONICET), Universidad Nacional de Misiones (UNaM), Jujuy 1745, 3300 Posadas, Misiones Argentina

**Keywords:** Cytogenetics, Genetic markers, Evolution, Genetics

## Abstract

Capuchin monkeys (genera *Cebus* and *Sapajus*) show a wide range distribution, from Honduras to Argentina. The aim of this work was to evaluate the genetic and phenotypic variability of captive specimens putatively belonging to *S. cay* (SCY) and *S. nigritus* (SNI) at their southernmost distribution limit. Forty-four individuals held in five captive centers from Argentina were analyzed based on external morphology, karyology and DNA sequences of mitochondrial control region (mtDNA-CR). Three morphotypes associated with their probable geographical origin in SCY and a single morphotype in SNI were found. For SCY we could associate each morphotype with the most frequent karyotype. SNI showed a single phenotype and a homogenous karyotype. Heterochromatin showed geographical patterns within species. A 515-bp mtDNA-CR fragment was sequenced, defining fourteen haplotypes at 59 polymorphic sites. A network constructed with our 14 haplotypes and other 77 from *S. apella*, *S. macrocephalus*, *S. cay* and *S. nigritus* from bibliography revealed some phylogeographic signals. Our SCY and SNI samples rendered four groups that differed in multiple mutational steps, with SCY being more similar to *S. apella* than to *S. macrocephalus*. Also, we identified two genetic divergent SCY groups: samples from NOA and from NEA with high mitochondrial diversity. Our results highlight the relevance of using complementary genetic tools throughout the distribution ranges of SCY and SNI for a better assessment of their diversity.

## Introduction

Capuchin monkeys, also known as caiararas or macacos-prego, are among the most widely distributed Neotropical primates (Platyrrhini). They inhabit almost all forested areas from Honduras and Nicaragua in Central America to the Argentinean provinces of Misiones, Salta, Jujuy, Formosa and Chaco in South America^1–3^. Capuchins display a remarkable diversity across their distribution range in terms of ecological, morphological, phenotypical (particularly the coat color), behavioral and genetic features at both inter- and intra-population levels. As a result, the taxonomy of this group has been repeatedly revised^4–9^.

Currently, capuchin monkeys are assigned to genera *Cebus* and *Sapajus* (gracile and robust capuchins, respectively)^10,11^. The species studied in the present work, *Cebus cay* and *Cebus nigritus*, were transferred to *Sapajus* by Lynch Alfaro et al.^11^. However, numerous studies on the taxonomy, evolutionary cytogenetics and molecular genetics of capuchins conducted over the last 40 years support the original classification of these monkeys into a single genus^12–19^. Notwithstanding this, in the present study we adopted the current taxonomic classification of this group to prevent misinterpretations of the results and to allow us to use available information on genetic variability.

**Figure 1:**
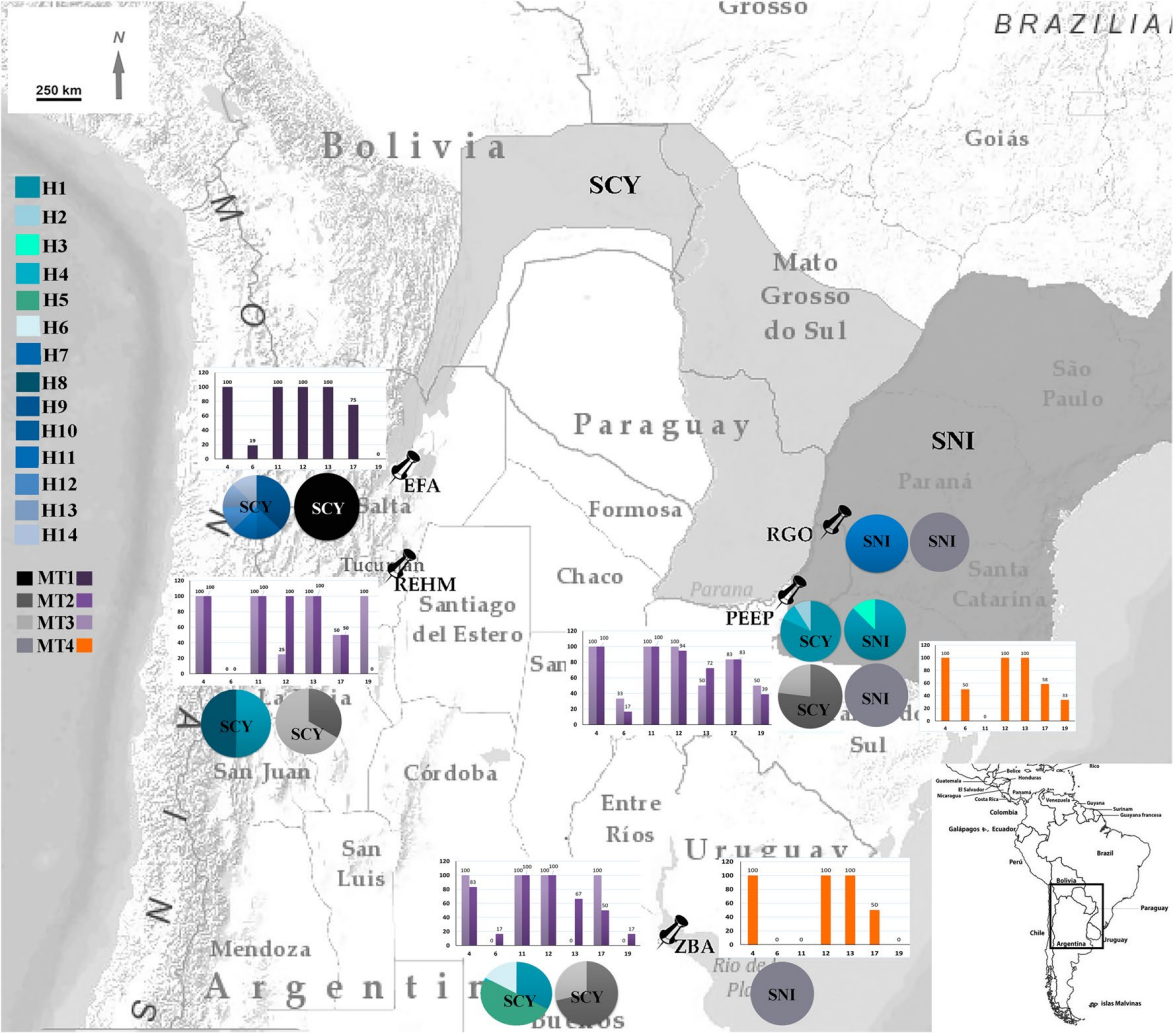
Map illustrating the southernmost natural distribution of capuchin monkeys in South America and location of the different Argentinian centers where samples were obtained. Modified from^59,60^ and UICN species distribution maps. *EFA* Estación de Fauna Autóctona (Salta), *REHM* Reserva Experimental Horco Molle (Tucumán), *ZBA* Zoológico de Buenos Aires, *PEEP* Parque Ecológico El Puma, *RGO* Reserva Güira Oga, *SCY Sapajus cay*, *SNI Sapajus nigritus*. Percent of heterochromatic blocks in different polymorphic chromosome pairs of each center are shown in bar diagram (purple scale and orange represent the chromosomal frequencies of each morphotype). Haplotype (H_) and morphotype (MT) frequencies in each center are shown in the pie charts and their size is proportional to sample size. The haplotypes and morphotypes are color-coded.

According to Rimoli et al.^20^, *Sapajus cay* (SCY) is resident and native in Brazil, Bolivia, Paraguay and Argentina. In Brazil can be found in the states of Mato Grosso and Mato Grosso do Sul (south, east, west and south-west). In Paraguay, *S. cay* occurs to the east of the Paraguay River and the distribution extends southward to the eastern part of the country, reaching into northwestern Argentina. It also occurs in southeast Bolivia, and northern Argentina (provinces of Jujuy, Salta, Formosa and Chaco). The distribution of *S. cay* forms a horse shoe shape, bordering the xerophytic Chaco of Argentina, Bolivia and Paraguay. In these countries, the species’ geographic limits are the Andes Mountains of Argentina and Bolivia to the west and the east of the Paraguay River, in Paraguay to the east; the confluence of the Paraguay and Parana Rivers might represent its south-easternmost limit^20^. *Sapajus nigritus* (SNI) occurs south of the Rio Doce, in the Brazilian states of Minas Gerais (south of the Doce River), São Paulo, Paraná, Santa Catarina and Rio Grande do Sul. In Argentina it is found at the northernmost tip of the Argentine province of Misiones (east of the Paraná River). The limits are the Doce River in the north, the State of Rio Grande do Sul in the south (that is not defined yet), the Atlantic Ocean in the east and the province of Misiones in Argentina in the west^21^. Lynch-Alfaro et al.^22^ suggests the presence of two *S. nigritus* subspecies: *S. n. cucullatus* in Santa Catarina and Rio Grande do Sul (Brazil), and Iguazú (Argentina); and *S. n. nigritus* in Minas Gerais, Espírito Santo, São Paulo and Rio de Janeiro (Brazil). The provinces of Salta and Chaco in northern Argentina and northern Rio Grande do Sul state in Brazil represent the southernmost distribution limit for SCY and SNI (Fig. 1).

Capuchin monkeys have particular genomic and chromosomal features: a conserved karyotype compared with that of the ancestral Neotropical primate, the largest amount of genetic information at the euchromatin level shared with humans, and large and conspicuous heterochromatic regions distributed throughout the karyotype. In capuchins, extracentromeric heterochromatin is genus-specific and shows intraspecific and interspecific variability. This makes the heterochromatin pattern a suitable marker for taxonomic identification, particularly for specimens of unknown geographical origin^19,23–26^.

Both species exhibit phenotypic variations mainly in coat and face coloration^1^. They are phylogenetically close species well suited to illustrate the most outstanding feature of capuchins’ genome, namely, the great variability in the amount and distribution of extracentromeric heterochromatin. Particularly, SCY has an extracentromeric heterochromatin block representing 75% of the q arm in chromosome 11, which is absent in SNI^27,28^.

Molecular genetic studies on arboreal primates have received increasing attention in the last years. Approaches using nuclear genetic markers and/or mitochondrial DNA sequences have contributed greatly to our understanding of phylogenetic, phylogeographic and biogeographic relationships among Neotropical primates^17,29–34^. Several studies addressing the spatial distribution of genetic lineages of the same or closely related species have focused on the analysis of the non-coding control region of the mitochondrial DNA (mtDNA-CR) or D-loop due to its high substitution rate and polymorphisms concentrated in the hypervariable region^35,36^. In particular, mtDNA-CR sequences from SCY and SNI have been examined to infer molecular phylogenies of capuchins or to identify *Sapajus* matrilineal haplotypes^32,33^.

Despite major advances in our knowledge of genomics of capuchin monkeys during the last decade, relevant information is still missing due to gaps in sampling. This is the case for their geographic distribution area in Argentina.

This study presents a holistic analysis of phenotypic, karyotypic and mtDNA-CR variation in 44 specimens belonging to two putative species of *Sapajus,* some of them with unknown provenance, housed at five centers in Argentina. The current study constitutes the first mtDNA-CR description in specimens from Argentina, the southernmost distribution limit of the genus. Chromosomal and phenotypic variability is analyzed considering new samples and approaches extending the findings from previous works^19,37^. It is also examined the contribution of the results here presented together with published information about wild and captive individuals of known species and origin can help understand the data from the uncertain provenance of captive individuals. Our results are expected to contribute to come up with a better understanding of the diversity and evolution of capuchin monkeys.

## Results

Genetic and phenotypic variability of captive specimens of robust capuchin monkeys putatively belonging to *Sapajus cay* (SCY) and *Sapajus nigritus* (SNI) were evaluated. Forty-four individuals held in five captive centers in Argentina were studied based on external morphology, karyology, and DNA sequences of the mitochondrial control region (mtDNA-CR) (Fig. 1; Table 1). The individuals were classified into four morphotypes (MT), based on external characteristics. Three of these (MT 1, 2 and 3) corresponded to SCY and the remaining one (MT 4) to SNI. A summarized description is provided as follows:

**Table 1:** Summary of the 44 analyzed *Sapajus* individuals kept in captivity in different Argentinean centers.

Institution	Collection number (§)	Provenance (∞)	
		Known	Unknown
Parque Ecológico “El Puma” (PEEP)^a^	SCY 674–676, 698, 699,706, 708–711, 729, 730, *736	Carmen del Paraná, Paraguay (708); Garupá, Misiones (710, 711)	674–676, 698, 699,706, 709, 729, 730
	SNI 677, 687,696, 697,*707, 712,734, 735	Oberá, Misiones (677, 712); Cruce Caballero, Misiones (687); Montecarlo, Misiones (696)	697, 734, 735
Reserva Privada Güira Oga (RGO)^b^	SNI 760	Puerto Iguazú, Misiones	
Reserva Experimental Horco Molle (REHM)^c^	SCY 763–765		763–765
Zoológico de Buenos Aires (ZBA)^d^	SCY 414, 634, 733, 737, *738, 739, 740, 741		414, 634, 733, 737, 739–741
	SNI 732		732
Estación de Fauna Autóctona (EFA)^e^	SCY 773, 775, 776, 777, 779, 780, 781, 782, 783, 784	Bolivia (773); Orán, Salta (775, 776, 781); Güemes, Salta (777); J.V. González, Salta (779); Aguaray, Salta (780); El Quebrachal, Salta (783)	782, 784
Total	44		

^a^Candelaria, Misiones (27° 27′ 39.7″ S–55° 44′ 38.5″ W).

^b^Puerto Iguazú, Misiones (25° 35′ 49.1″ S–54°34′ 43.1″ W).

^c^San Miguel de Tucumán, Tucumán (26° 48′ 32.1″ S–65° 13′ 14.8″ W).

^d^Ciudad Autónoma de Buenos Aires, Buenos Aires (34° 34′ 44.4″ S-58° 24′ 55.9″ W).

^e^Ciudad de Salta, Salta (24° 46′ 59.6″ S–65° 24′ 41.7″ W).

(§) Corresponds to the collection of our research group: Grupo de Investigación en Biología Evolutiva (GIBE). (∞) Origin of the individual regardless of whether or not the referred locality corresponds to the natural distribution of the species. *Captive-born individuals.

**Figure 2:**
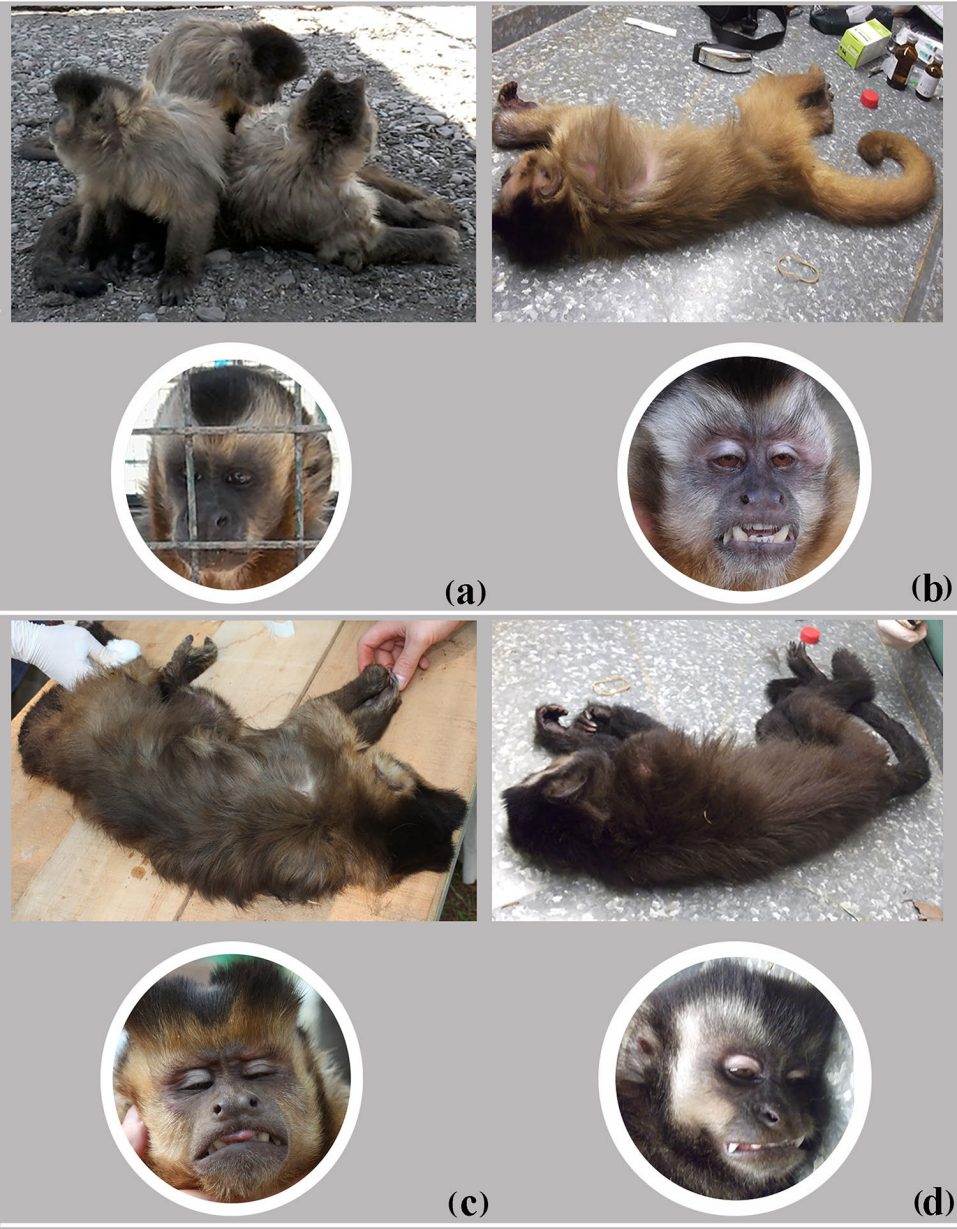
Typical phenotypes of the studied individuals. SCY Morphotype 1 (**a**). SCY Morphotype 2 (**b**). SCY Morphotype 3 (**c**). SNI Morphotype 4 (**d**). Photographs by M. Nieves.

- Morphotype 1 (MT 1; Figs. 1, 2a): it had a yellowish-white head, with black forehead and temples. The belly and chest were grayish or light brown, contrasting with the black crown and sideburns. There was a prominent dark dorsal stripe. The limbs were mainly dark brown to blackish and the upper arms were not lighter than the body. Individuals grouped in MT 1 were #773, 775, 776, 777, 779, 780, 781, 782, 783 and 784 from Estación de Fauna Autóctona (EFA), Salta.
- Morphotype 2 (MT 2; Figs. 1,  2b): The belly, chest, back and upper third of the arms were light yellow. Tail, limbs and back of head were dark brown. Shoulders were lighter than the rest of the body. Individuals grouped in MT 2 were #634, 737, 738, 739, 740 from Zoológico de Buenos Aires (ZBA), Buenos Aires; #675, 676, 706, 708, 709, 710, 711, 729, 730, 736 from Parque Ecológico El Puma (PEEP), Misiones and #765 from Reserva Experimental Horco Molle (REHM), Tucumán.
- Morphotype 3 (MT 3; Figs. 1, 2c): The belly and chest were light brown. The back, limbs and tail were dark brown; the top of the head and crests were blackish. Individuals grouped in MT 3 were #733 and 741 from ZBA, Buenos Aires; #674, 698 and 699 from PEEP, Misiones and #763 and 764 from REHM, Tucumán.
- Morphotype 4 (MT 4, Figs. 1, 2d): The coat varied from dark brown to blackish over the body, with no (or very vague) dorsal stripe. Its face was whitish and had inconspicuous sideburns. The limbs were darker than the body, usually blackish. Pointed crown tufts in adults, if present, wear away with age. The individuals grouped in MT 4 were #677, 687, 696, 697, 707, 712, 734 and 735 from PEEP, Misiones¸ #760 from RGO, Misiones and #732 from ZBA, Buenos Aires.

**Figure 3:**
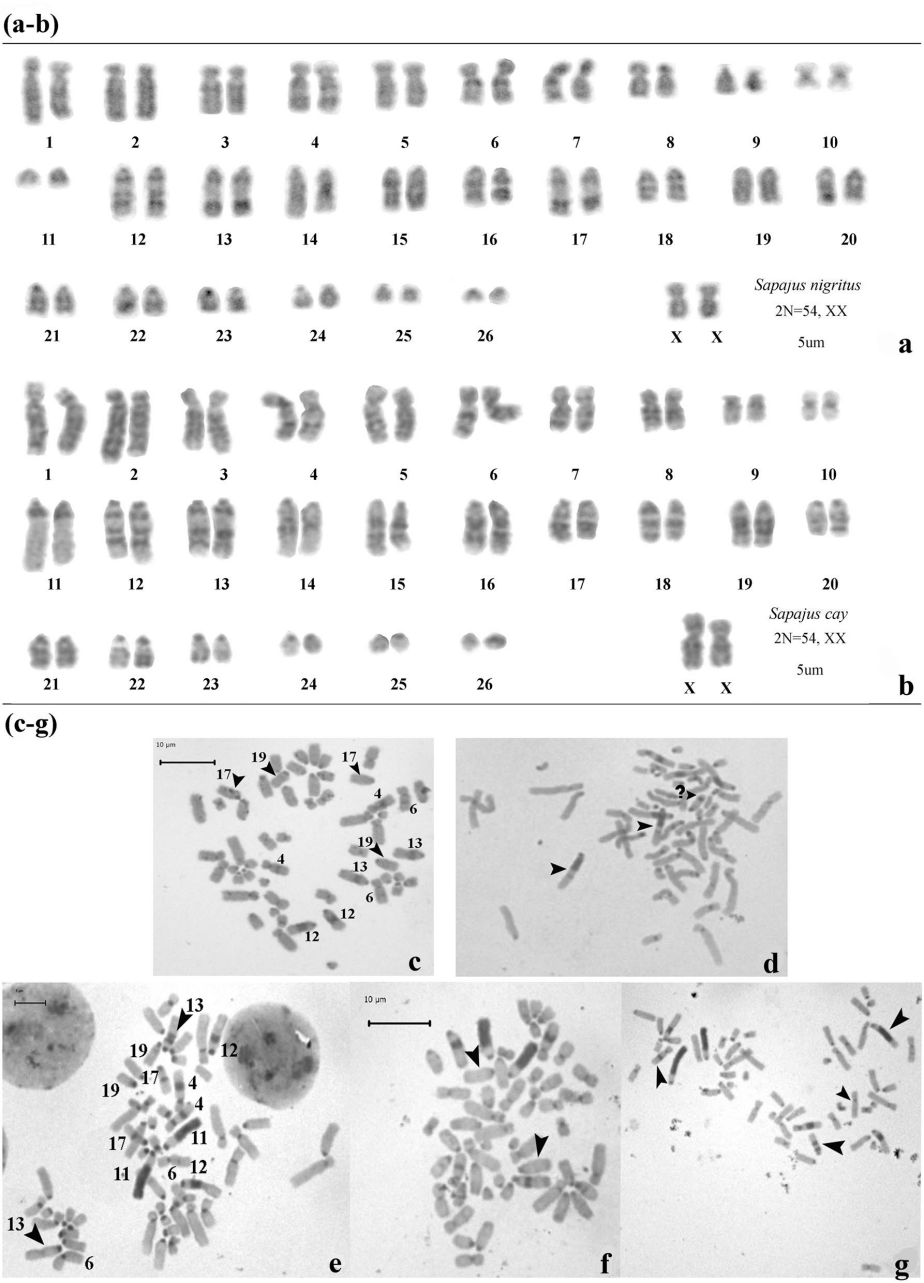
G and C banding patterns and polymorphisms found among the studied individuals. (**a**,**b**) G-banded patterns observed in SNI and SCY respectively. (**c**–**g**) C-banded metaphases illustrating different heterochromatin polymorphisms found in both species, highlighted with arrows. (**c**) Polymorphisms in chromosomal pairs #13, 17 and 19 of *Sapajus nigritus* and a paracentric inversion resulting in a new heterochromatic band. (**d**) (?) shows an undescribed band. (**e**–**g**) Polymorphism in chromosomal pair #13 of *Sapajus cay.*

Karyological diagnosis confirmed the taxonomic status in 77.27% (34/44) of the specimens studied, of which 27 were assigned to SCY and 7 to SNI. In the remaining 10 individuals, lymphocyte culture yielded non-evaluable results (Table 2). We were able to discriminate between polymorphisms for C + band (i.e., absence/presence of a C + band or differences in block size) and polymorphisms for structural rearrangements involving C + bands. All the individuals showed the centromeric heterochromatic band in the entire complement. Blocks of pairs #4, 11 and 12 were present in almost all metaphases from SCY individuals, while the block of pair #11 was absent in all SNI individuals. Figure 3 shows a typical G-band (Fig. 3a,b) and heterochromatin patterns of both species including examples of heterochromatic polymorphisms observed. In addition, Fig. 1 illustrates for most centers sampled, bar diagrams with frequencies of different C + bands in each chromosomal pair with extracentromeric heterochromatin. In both species, chromosomal pairs #6, 13, 17 and 19 were polymorphic for the presence of C + bands detected in homozygous and heterozygous condition. Pair #19 was the most polymorphic for both species. In SNI (Fig. 3c), the C + band was absent in one of the homologues of 4/7 (57.2%) individuals and in both homologues of 3/7 (42.8%) individuals, while in SCY, the C + band lacked in one of the homologues of 3/27 (11.11%) specimens and in both homologues of 16/27 (59.3%) specimens. Chromosomal pair #17 was the second most polymorphic for SNI individuals, of which 5/7 (71.4%) lacked the C + band in one of the homologues and 1/7 (14.3%) in both of them. For SCY, the second most polymorphic pair was #6, with 3/27 (11.11%) individuals lacking the C + band in one homologue or showing C-band size heteromorphism, and 17/27 (62.96%) specimens without the C + band in both homologues. In regard to the chromosomal pair #13, in SCY, absence of C + band in one homologue and C + band size heteromorphism were found in 7/27 individuals (25.92%) (Fig. 3e), while there was no C + band in either homologue in 4/27 (14.81%) individuals (Fig. 3f). In addition, one SCY individual showed a paracentric inversion resulting in two C + bands (Fig. 3g, see arrows) and 2 SNI individuals showed additional C + bands in chromosomal pairs #12 and 13 (Fig. 3d, see arrows).

**Table 2:** Summary of morphological, karyological and molecular data obtained in the present study.

Species	Morphotype (MT)	Institution	Collection number	Cytogenetic studies										Molecular studies
				BG	BC								Observations	mtDNA-CR
					C+	4	6	11	12	13	17	19		Haplotype ID
SCY	MT 1	EFA	773	√	++	++	+−	++	++	++	+−	−−		H_9
SCY	MT 1	EFA	776	√	++	++	−−	++	++	++	++	−−		H_10
SCY	MT 1	EFA	779	√	++	++	−−	++	++	++	+−	−−		H_9
SCY	MT 1	EFA	781¥	NA										H_12
SCY	MT 1	EFA	782	√	++	++	−−	++	++	++	++	−−		H_13
SCY	MT 1	EFA	784¥	NA										H_9
SCY	MT 1	EFA	780	√	++	++	−−	++	++	++	++	−−		H_11
SCY	MT 1	EFA	783	√	++	++	++	++	++	p **	++	−−	** Pa. Inv	H_14
SCY	MT 1	EFA	775	√	++	++	−−	++	++	++	++	−−		NA
SCY	MT 1	EFA	777	√	++	++	−−	++	++	++	−−	−−		NA
SCY	MT 2	PEEP	675	√	++	++	+−	++	++	++	+−	+−		H_1
SCY	MT 2	PEEP	676	√	++	++	+−	++	++	+−	+−	+−		H_2
SCY	MT 2	PEEP	706	√	++	++	−−	++	+−	+−	++	+−		H_1
SCY	MT 2	PEEP	708	NA										H_1
SCY	MT 2	PEEP	709	√	++	++	−−	++	++	+−	+−	+−		H_1
SCY	MT 2	PEEP	710	√	++	++	−−	h	++	++	++	+−		H_4
SCY	MT 2	PEEP	711	√	++	++	−−	h	++	++	++	−−		H_1
SCY	MT 2	PEEP	729	√	++	++	−−	++	++	−−	++	−−		NA
SCY	MT 2	PEEP	730	√	++	++	+−	++	++	++	++	+−		NA
SCY	MT 2	PEEP	736	√	++	++	−−	++	++	++	++	+−		H_1
SCY	MT 2	REHM	765	√	++	++	−−	h	++	++	+−	−−		H_1
SCY	MT 2	ZBA	634	NA										H_1
SCY	MT 2	ZBA	737	√	++	++	−−	h	++	+−	+−	−−		H_1
SCY	MT 2	ZBA	738	√	++	+−	+−	++	++	p **	+−	−−	** Pa. Inv	H_5
SCY	MT 2	ZBA	739	√	++	++	−−	h	++	+−	+−	+−		H_5
SCY	MT 2	ZBA	740	NA										H_5
SCY	MT 3	PEEP	674	√	++	++	+−	++	++	++	++	+−		H_1
SCY	MT 3	PEEP	698	P *	++	++	+−	++	++	−−	+−	+−	* 13 & 16 polymorphic	H_1
SCY	MT 3	PEEP	699	√	++	++	−−	++	++	+−	++	+−		H_1
SCY	MT 3	REHM	764	√	++	++	−−	++	+−	++	+−	P **	** Pa. Inv	H_8
SCY	MT 3	REHM	763	√	++	++	−−	++	−−	++	+−	P **	** Pa. Inv	NA
SCY	MT 3	ZBA	733	√	++	++	−−	++	++	−−	++	−−		NA
SCY	MT 3	ZBA	741	NA										H-6
NA	−−	ZBA	414	NA										H_1
SNI	MT 4	ZBA	732	√	++	++	−−	−−	p **	++	+−	−−	** Pa. Inv	NA
SNI	MT 4	PEEP	677	p *	++	++	+−	−−	++	p **	+−	+−	* Pe. Inv. 13, ** Pa. Inv	H_1
SNI	MT 4	PEEP	687	NA										H_1
SNI	MT 4	PEEP	696	√	++	++	++	−−	++	++	+−	−−		H_1
SNI	MT 4	PEEP	707	√	++	++	+−	−−	++	++	+−	+−		H_1
SNI	MT 4	PEEP	712	√	++	++	+−	−−	++	++	++	+−		H_1
SNI	MT 4	PEEP	734	NA										H_1
SNI	MT 4	PEEP	735	√	++	++	−−	−−	++	++	+−	+−		H_1
SNI	MT 4	PEEP	697	√	++	++	+−	−−	++	++	+−	−−		H_3
SNI	MT 4	RGO	760	NA										H_7

√, normal G-pattern; p, polymorphism; + −, presence/absence; h, size heteromorphism; NA, not available. Pe. Inv, pericentric inversion; Pa. Inv, paracentric inversion. Chromosomal data from Nieves et al., 2017. ¥ New analyzed individuals.

The mtDNA-CR analysis indicated that the designed specific primers allowed the successful amplification of a 515 bp fragment for each of the 44 specimens analyzed. A total of 37 (84%) good-quality individual sequences (9 for SNI and 28 for SCY) were obtained, used in subsequent analyses and submitted to GenBank (KJ737380-KJ737416). The absence of nuclear mitochondrial DNA segments was confirmed by the presence of single bands in PCR amplifications and single peaks in mtDNA-CR sequences. One sample from ZBA (#738) was excluded from the variability analysis because it belonged to an offspring of specimen #740. When considering both species; we identified 14 haplotypes defined by 59 variable sites. We detected 40 transitions and 1 transversion in SCY, and 33 transitions and 2 transversions in SNI (Table S1). Haplotype 1 (H_1) was the most frequent in both species and the only haplotype shared between them. The remaining haplotypes were similar in frequency; two of them were only detected in SNI whereas 11 haplotypes were only identified in SCY (Tables 2, 3). Substantial variation was found within SCY, with a large number (9/12) of unique haplotypes. According to these results, SCY showed higher levels of genetic diversity—as evaluated by haplotype diversity (*h*) and nucleotide diversity (*π*)-compared with SNI (Table 3). Within SCY, the morphotype 1 with the highest haplotype diversity and moderate nucleotide diversity was detected in EFA indicating a high number of closely related haplotypes in Salta (NOA). Both morphotype 3 with highest nucleotide diversity or morphotype 2 with the lowest genetic indices were found in both NOA and NEA regions.

**Table 3:** Absolute haplotype frequencies and genetic diversity indices (haplotype diversity (h) and nucleotide diversity (π)) with their standard deviation for SCY and SNI and for different morphotypes within SCY using mitochondrial DNA control region (mtDNA-CR) sequence data.

Haplotype ID	SCY				SNI
	Morphotype1	Morphotype 2	Morphotype 3	Not available Morphotype	Morphotype 4
H_1		9	3	1	7
H_2		1			
H_3					1
H_4		1			
H_5		2			
H_6			1		
H_7					1
H_8			1		
H_9	3				
H_10	1				
H_11	1				
H_12	1				
H_13	1				
H_14	1				
Haplotype diversity (*h*)	0.893 ± 0.111	0.571 ± 0.132	0.700 ± 0.218	–	
Nucleotide diversity (π)	0.015 ± 0.009	0.013 ± 0.007	0.0278 ± 0.017	–	
Haplotype diversity (*h*)	0.766 ± 0.084				0.417 ± 0.191
Nucleotide diversity (π)	0.023 ± 0.012				0.015 ± 0.008

We also included mtDNA-CR reference sequences of SCY, SNI, *S. apella* (SAP) and *S. macrocephalus* (SMA) (two nearby species) from GenBank (see details in Material and Methods section) for a more comprehensive genetic diversity analysis of the species analyzed here at their southernmost distribution limit. Three of the four SCY reference sequences from Brazil and Paraguay matched H_1, while the remaining one correlated with a haplotype that could not be attributed to any of the species under study (identified as HC). The analysis of the six reference sequences of SNI revealed five different haplotypes, which were not shared with the species analyzed here (identified as HN1, HN2, HN3, HN4 and HN5). The examination of twenty-six sequences of SMA identified twenty-four haplotypes whereas the study of sixty-three sequences of SAP distinguished forty-eight haplotypes. Only one reference sequence of SMA matched with the H_5 detected in SCY in the present paper.

**Figure 4:**
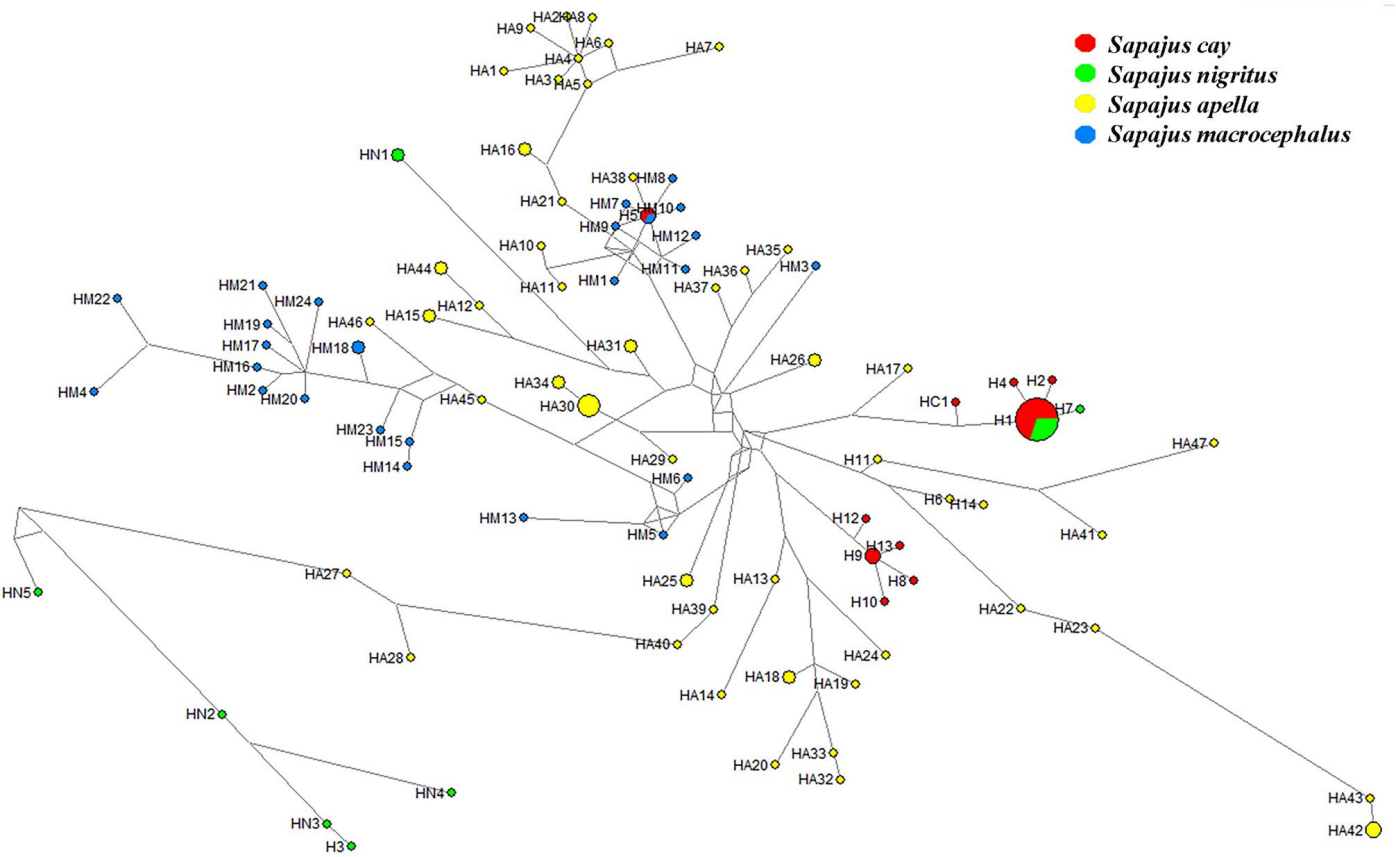
Median-joining haplotype network based on the 515 bp mtDNA-CR sequence from 36 specimens of *S. nigritus* and *S. cay* and published available sequences from these species and other nearby capuchin species, *S. apella* and *S. macrocephalus* (namely HC1 for *S. cay*, HN1-HN5 for *S. nigritus*, HA1-HA45 for *S. apella* and HM1-HM24 for *S. macrocephalus* see “Material and methods” section). The size of the circles (haplotypes) is proportional to the number of specimens sharing a given haplotype. Branches are proportional to the mutation number. *Sapajus cay* individuals’ haplotypes are represented in red, *S. nigritus* ones in green, *S. apella* in yellow, and *S. macrocephalus* in blue.

In general, the median-joining network constructed using both haplotypes identified here and those from reference samples clustered the *Sapajus* haplotypes according to the putative species (Fig. 4). SCY and SNI haplotypes were included in four groups that differed in multiple mutational steps. One of them (Group A) included the most frequent haplotype (H_1), from which derived two singly occurring haplotypes unique to SCY housed in PEEP (H_2, H_4) and one singly occurring haplotype unique to SNI housed in RGO (H_7) as well as one previously detected haplotype in SCY from Mato Grosso, Brazil (HC1). Two groups (Groups B and C) included only haplotypes detected in SCY. Group B comprised the H_9 from which four closely related singletons housed in EFA and REHM (H_8, H_10, H_12, H_13) emerged. Group C included related haplotypes housed in ZBA and EFA (H_6, H_11, H_14). As a general factor, SCY haplotypes identified in the present paper were more similar to SAP than to SMA haplotypes, indeed, some haplotypes for NOA (H6 H11 H14) were most like SAP haplotypes from south Brazil.

The remaining group (Group D), different from any other haplotype group, included related haplotypes of SNI: H_3 haplotype detected in the present contribution and four related haplotypes (HN2, HN3, HN4, HN5) detected in reference samples from southern Brazil and Northern Argentina. Finally, two haplotypes identified here were distant from the other ones. One of them, H_5 belonging to 2 SCY specimens from ZBA matched to a SMA reference sequence. From it emerge some others SMA haplotypes in the network. The other one, HN1 corresponding to a SNI sample reference, clustered with SAP haplotypes from which diverged in many mutational steps.

## Discussion

Over the last years, the diversification and radiation of capuchin monkeys have been analyzed and different biogeographical hypothesis were tested. However, the results of these studies were obtained only with molecular datasets, with less attention paid to karyotype and morphological variation. This is particularly certain for the two species studied herein, which were transferred to *Sapajus* and are located at the southernmost distribution limit of this genus i.e.^31,33,34,38^. In this context, the present contribution was centered in characterizing captive specimens, some of them with unknown provenance, of putative *Sapajus cay* and *Sapajus nigritus* analyzing simultaneously molecular, chromosomal and morphological datasets as complementary sources to achieve a more accurate evaluation of their variability.

The presence of conspicuous heterochromatic blocks corresponding to repeated sequences distributed throughout the karyotype is a distinctive cytogenetic feature of capuchin monkeys. Previous results of Nieves et al.^19^ proposed that for SCY and SNI the most polymorphic chromosome pairs were #6, 17 and 19: in SNI the highest frequencies were observed for pairs 17 and 6, while in SCY for pairs 6 and 17. Chromosome pair 19 showed a similar but lower percentage of heteromorphism in both species. Also, it was concluded that there was only a weak relationship between geographical origin and heterochromatin pattern at the intraspecific level. In the present contribution adding new individuals, reanalyzing the published data and considering the provenance of several samples (which was unknown in the previous study), it was showed new evidence to distinguish species and to characterize the centers, within species, in relation to extracentromeric heterochromatin distribution.

According with previous studies, the heterochromatin pattern observed in the biggest acrocentric pair (#11) is a marker for taxonomic diagnosis in capuchin monkeys. All SCY individuals were characterized for the presence of the block of pair #11 in double doses although some size heteromorphisms could be observed whereas SNI individuals did not exhibited the extracentromeric heterochromatin block. In general, karyotype differences based on heterochromatic blocks do not constitute reproductive barrier because heterozygotes for such blocks do not affect the fertility of the carriers^39^. In those cases, karyotype differentiation may accompany speciation rather than promote it. In addition, some common chromosome features detected within centers, i.e., SCY Individuals from Northwest EFA (Salta center), most of them from Salta (Argentina) or Bolivia, exhibited low level of chromosome polymorphisms. On the other hand, individuals from PEEP, some of them from Paraguay, exhibited high level of polymorphism. Similar results were observed in individuals from ZBA (Buenos Aires) and REHM (Tucuman) with unknown provenance although probably from several origins. In regard to SNI, in both centers ZBA and PEEP, the individuals exhibited similar and moderately polymorphic heterochromatin patterns.

The analysis of phenotype demonstrated that samples of Northwest SCY individuals with inferred and assigned origin in Bolivia and Salta (EFA) also exhibited phenotype homogeneity (all individuals corresponded to MT 1) whereas monkeys from PEEP, ZBA and REHM exhibited either of two morphotypes (i.e., MT 2 or 3). In regard to SNI, the comparison of our results with those obtained by^26,27,40^ revealed that only the SNI individuals studied here presented the phenotype blackish body pelage and a lighter face (MT4).

Recently, Penedo et al.^26^ studied 26 captive individuals belonging to *Sapajus*, in Rio de Janeiro, Brazil. By analyzing the C-banding patterns and their pelage coloration these authors classified the animals into at least four large classes. Interestingly, they found that each C-banding pattern was associated with at least two phenotypes concerning pelage coloration. In our sample of SCY, even considering the occurrence of three morphotypes and different levels of chromosomal polymorphisms, we were able to discriminate and assign each morphotype with the most frequent karyotype (Fig. 1 bar diagrams and MT pie charts). In regard to SNI, the comparison of our results with those obtained by^26,27,40^ revealed that only the SNI individuals studied here presented the phenotype blackish body pelage and a lighter face, and a karyotype in which chromosomal pair #11 lacks the extracentromeric heterochromatin block described by^28^.

The results mentioned above may indicate higher phenotypic and karyotypic intra- and inter population variation in SNI from Brazil^26^ than from Argentina. A possible explanation may be related to the occurrence of polytypic populations of Brazilian SNI living in sympatry with a mixture of karyotypes and phenotypes in hybrid areas. Further studies involving a complete karyological characterization of SNI from their natural range in Brazil -especially populations close to Misiones province in Argentina, such as those in Santa Catarina, Paraná and Rio Grande do Sul- and a subsequent comparison with SNI from Argentina are needed to establish this point conclusively.

The analysis of the 515 bp mtDNA-CR sequence revealed that the studied SCY individuals exhibited the highest level of genetic diversity in terms of haplotype and nucleotide diversity. This result may be explained by the fact that the samples identified as SCY comprised individuals from different geographic origins. The samples assigned to SNI, which included a smaller number of individuals whose putative geographical origin was restricted to a single province, showed lower levels of variability, with at least three haplotypes. Comparing the centers regarding haplotype diversity, EFA in the Northwest appeared as the most diverse (in contrast to what is observed at chromosome and morphology level), whereas PEEP, REHM and ZBA exhibited a low number of haplotypes but exhibited higher phenotype and chromosome variation. Current results pointed out that H_1 was identified as the most frequent haplotype in both studied species and the reference samples of SCY.

The network constructed with haplotypes identified here and reference sequences showed some phylogeographic structure suggesting the existence of at least four groups of haplotypes with one of them (Group A) being the most frequent in the Northeastern Region of Argentina (NEA) and which include SCY and SNI individuals. Groups B and C were only observed in SCY individuals from the relatively closely located centers in Salta and Tucumán, suggesting that these haplotype groups may be representative of the Northwestern Region of Argentina (NOA). SCY Haplotypes from NOA exhibited major similarity with SAP from relatively near geographic area. Group D clustered haplotypes detected in SNI, one haplotype identified in PEEP (Misiones) (H_3) and several haplotypes described previously in Brazil (HN2, HN3, HN4, HN5). It is striking the position in the network of an isolated haplotype, H_5, belonging to two *S. cay* individuals from JZBA of unknown origin and one *S. macrocephalus* from literature. For this case, it could be proposed at least two hypotheses, (1) that those animals are hybrids between *S. cay* and *S. macrocephalus,* or (2) that they are indeed *S. macrocephalus*. However, to corroborate this second option, we do not have the *S. macrocephalus* karyotype as it has not been described (or at least it has not been published yet). Another isolated haplotype (HN1) in the network belonged to a sample which formerly was assigned to SNI but recently were reassigned to *S. robustus*^9^ explaining the divergence of sequences. HN2 haplotype corresponds to a sample which was also reassigned to *S. robustus* but maintain a clear relationship with SNI haplotypes.

No previous information about *Sapajus*’ mtDNA variation in north Argentina was reported up to date. Our analysis of mt DNA-CR of *S. cay* also showed two divergent groups: samples from NEA with assigned or probable origin in Paraguay, related to haplotype (HC)^33^ assigned to southern Mato Grosso (Brazil) and close to a *S. apella* haplotype (HA17) from northern Amazon; and individuals from NOA with assigned origin in Salta (Argentina) and Bolivia with high mitochondrial genetic diversity and more related to *S. apella* haplotypes from nearby regions than to the other *S. cay* groups. Some studies based on sequence data for other genes (Cyt b and COI genes), described mt DNA variation of *S. cay* from Southeastern Paraguay and Mato Grosso do Sul (south, east, west and south-west) (Brazil)^9,33^. Further studies must consider approaches using the same mtDNA marker and examining all localities where *S. cay* was detected to get a better knowledge about mtDNA genetic variability in this species.

Three haplotypes were detected in SNI: H_1, identified also in samples of SCY from our analysis and previous ones; H_7, a haplotype very close to H_1 and; H_3, a very divergent haplotype that was not identified until now and very close to SNI haplotypes previously detected. The existence of zones of contact between both species suggests that hybridization may have been frequent during the evolution of the lineages^9,10^. With our new finding in mind, one is tempted to propose that individuals identified as SNI can be hybrids between both species analyzed here. Still, our results revealed that all individuals identified as SNI exhibited the same karyotype and morphotype. One likely scenario to explain our results may be related with a past hybridization event occurred many generations ago and this event is still retained in mt DNA SNI samples from the present study as was proposed for macaques^41^. However, this hypothesis rests on very low number of samples. It is necessary to perform a wider analysis involving chromosome, phenotype and molecular approaches to gain deeper in sight into the variability in this taxon.

The present study constitutes the first description of mtDNA-CR in SCY and SNI individuals at the southernmost distribution limit of the genus. A trend toward greater nucleotide variability for SCY than for SNI was observed in our limited sample. Likewise, cytogenetic variability was higher for SCY than for SNI in terms of polymorphisms for extracentromeric heterochromatin and for structural rearrangements. In the case of SNI, more studies are needed to conclusively determine the level of genetic diversity and population structure. We recognize the need for new morphological, chromosomal, and molecular analyses for subsampled and unexplored geographic regions of both species distributions to improve our knowledge about intraspecific and interspecific variation.

The analysis of the mtDNA-CR made in our study proved to be an important tool for an accurate identification of species belonging to genus *Sapajus*, and useful for both in situ and ex situ management. In general terms, under captivity conditions, it seems advisable to group together animals with similar polymorphisms at the chromosomal and mtDNA-CR levels, since they may come from the same population.

## Conclusions

- The present study constitutes the first description of mtDNA-CR in SCY and SNI individuals at the southernmost distribution limit of the genus.
- A phylogeographic approach distinguished mtDNA lineages of *S. cay* from northwestern and northeastern Argentina
- The analysis of the mtDNA-CR made in the present phenotypic and genetic study proved to be an important tool for the accurate identification of species belonging to genus *Sapajus*, and useful for both in situ and ex situ management.

## Material and methods

All animal work was approved by the Ethical Committee of the Argentine Society for the study of Mammals (SAREM).

The study was carried out in compliance with the ARRIVE guidelines (https://arriveguidelines.org).

### Animal housing and management

Capuchins were housed in five Institutions located in the northwest (REHM and EFA), northeast (PEEP and RGO) and center-east (ZBA in the capital city) of Argentina (Table 1), where they were maintained in compliance with local regulations. Such institutions were zoos and/or rescue centers for animals confiscated from illegal trafficking and possession. Except for the EFA, they were opened to the public for visiting. The enclosures differed according to the general collection plan of the institutions. In REHM, EFA, PEEP and RGO, animals were kept in outdoor wire-mesh enclosures with soil floor and a roof, while in ZBA they were in an open enclosure located on a large island surrounded by moat of water and provided with several shelters. In the institutions where individuals of both species were found, PEEP and ZBA, they were always housed in separate enclosures, following the phenotypic description of^1,13^ for their separation. In all the institutions, newly arrived animals were isolated in a quarantine area for 10–20 days and then transferred to an enclosure close to the established collection, thus allowing for a gradual transition. In general, primates were grouped according to their age, size of the resident troop and size of the enclosure. They were fed at least once daily a balanced and varied diet containing seasonal fruits, vegetables and seeds, and red and white meats. Water was available ad libitum. Drinkers and feeders were cleaned and disinfected daily and enclosures at least every other day. Vitamin supplementation was performed at least once a week and deworming at least every six months. Animals were provided environmental and behavioral enrichment (with social, occupational, motor and sensory stimuli) based on their biological and behavioral requirements. General health status was sporadically assessed by veterinarians through the analysis of feces, urine and blood.

To obtain the blood samples used in our study, the veterinarians of each institution sedated the animals with Ketamine in their enclosures and then transferred them to the operation room, where they were placed on a stretcher and were administered maintenance anesthesia. After collection procedures, the animals were monitored until complete anesthetic recovery. The accommodation and maintenance of the individuals followed the welfare standards proposed by the code of ethics of ALPZA and WAZA (Latin American Zoo and Aquarium Association, 2018, https://www.alpza.com/herramientas; World Association of Zoos and Aquariums, 2018, http://www.waza.org/en/site/conservation/code-of-ethics-and-animal-welfare). The well-being and survival of all the specimens involved in this study represented a high priority and therefore euthanasia was not carried out.

### Phenotypic diagnosis

The different pelage coloration patterns and its variation among the individuals studied were analyzed based on data from Nieves and Mudry^37^ and re-evaluated (Table 2). Each specimen was photographed and assigned to either one of the two suspected species based on external characteristics, according to^1,13^ as follows:

- SNI (black capuchin): It has a very dark brown or gray, even blackish, pelage; with no (or very vague) dorsal stripe, the face is white and contrasts with the color of the body. The sideburns are poorly distinct; the limbs are darker than the body, usually blackish, presence of tufts on the crown, which are pointed in adults but become worn away with age.
- SCY (yellow-bearded capuchin): It has a yellow to white head with black crown and sideburns, which contrast with the light-colored body; presence of a prominent dark dorsal stripe; limbs mainly dark to blackish; upper arms not lighter than body; the underside is yellowish or reddish, often overlaid with black.

### Blood sample collection

Whole blood samples were collected from 44 captive individuals held in five Argentinean centers (Table 1; Fig. 1). After anesthetizing animals with ketamine (2 mg/kg weight), 2–5 ml of whole blood were obtained by venipuncture with disposable syringes and then transferred into two sterile tubes, one containing heparin (Sobrius, 500UI/ml) for cytogenetic studies, and the other EDTA (5% w/v) for molecular analyses.

### Karyological diagnosis

The patterns of chromosome variation were analyzed using the observations of the present work and data from previous studies (Table 2). To confirm the species status of each specimen, cultures of peripheral blood lymphocytes were used to apply G- and C-banding following^42^.

After the implementation of the C-banding protocol, at least 10 mitotic metaphases were analyzed per individual, with a particular focus on chromosome pairs having extracentromeric heterochromatin blocks and possessing structural rearrangements i.e. #4, 6, 11, 12, 13, 17, and 19.

The banding patterns thus obtained were compared with those already reported in the literature for *Cebus and Sapajus* spp*.*: *Cebus albifrons*^43^; *S. apella*^44^; *C. capucinus*^45^; *C. paraguayanus (S. cay)*^12^; *S. nigritus*^28^; *C. nigrivitattus (C. olivaceus)*^46^; and *S. xanthosternos*^47^.

### Molecular analyses

DNA extraction was carried out following^48^. For the amplification of the mtDNA-CR a new primer pair was designed using the Primer3 v. 0.4.0 software^49^. In addition, a sequence from a SNI sample previously obtained with the universal primer pair developed by^50^ (data not shown) was used: LD: CNI555 5′-GGCATACACAATTCTTTTCCTA-3′ and HD: CNI555 5′-ACCCTATGCATCCAGTGACG-3′. Polymerase chain reaction (PCR) amplifications were carried out in a 9600 Perkin Elmer thermocycler under the following conditions: 30 cycles of 93 °C for 1 min, 60 °C for 1 min, and 72° C for 3 min. PCR reactions were performed in a final volume of 25 μl containing 2 μl of DNA template (100–150 ng), 1 × PCR buffer with (NH4)_2_SO4 (Fermentas), 1.5 mM of MgCl_2_, 0.2 μM of each primer; 0.2 mM of each dNTP; and 0.5 U Taq polymerase (Fermentas).The PCR products were electrophoresed in 2% agarose gel with 0.5ug/ml of Ethidium Bromide (BIO-RAD) and visualized by an ultraviolet transilluminator. For each analyzed individual, forward and reverse nucleotide sequences were obtained by capillary electrophoresis using the sequencing facility of the Department of Ecology, Genetics and Evolution, FCEyN, University of Buenos Aires. Sequences were aligned and edited using BioEdit v.7.0.5.3^51^ and Clustal X v.1.8^52^. Variable nucleotide sites and haplotypes were identified using GenAlEx v.6.1^53^. Arlequin v3.11^54^ was used for computing estimates of nucleotide diversity (π) and haplotype diversity (h) according to^55^.

To determine the mtDNA-CR diversity of *S. cay* and *S. nigritus* in a broad context, we included the sequences from Argentina analyzed herein and the GenBank reference sequences of these species from Paraguay and Brazil. We used the following reference accession numbers: MF472524, MF472525^31^, KX592678^32^ and KY173186^33^ for the analysis of SCY; and KY173226, KY173227, KY173230^33,34^, MF472523, MF472522^31^ and KX592680^32^, for the analysis of SNI. Although the sequence KX592678 is 14 bp shorter than the 515-bp fragment analyzed here, it was included because there are no variable nucleotide sites in the missing portion. Finally, we excluded the sequence JQ317617 from^56^ because it is 52 bp shorter than the analyzed 515 bp-fragment and the missing portion contains variable sites.

Haplotype relationships were estimated using the sequences of *S. cay* and *S. nigritus* mentioned above and the GenBank reference sequences (which include the same DNA fragment analyzed here) from other nearby capuchin species, *S. apella* and *S. macrocephalus*. We considered the reference accession numbers KX756240.1, KX592677.1^32^; KY173143.1-KY173160.1, KY173162.1-KY173184.1^33^; MF472455.1-MFA72470.1, MF472483.1, MFA472499.1, MF472512.1-MFA472513.1^9^ for *S. apella* and KX592679.1^32^; KY173200.1-KY173213.1, KY173215.1-KY173225.1^33^ for *S. macrocephalus.*

Sequence alignments were performed with BLAST^57^ and a median joining network (MJN) implemented in NETWORK 5.0.1.1^58^ was constructed.

## Supplementary information

Supplementary information.
