# Supplementary material for: Assessment of genetic variability in captive capuchin monkeys (Primates: Cebidae)
Source: Sci Rep. 2021 Mar 31;11:7306. doi: 10.1038/s41598-021-86734-w (PMC8012615; doi:10.1038/s41598-021-86734-w)
Supplement: Supplementary file 1 — Supplementary information. [file 41598_2021_86734_MOESM1_ESM.docx]

**Supplementary material**

**Assessment of genetic variability in captive capuchin monkeys (Primates: Cebidae)**

*Mariela Nieves^*a, b^, María Isabel Remis^b,c^, Carla Sesarini^a^, Diana Lucrecia Hassel^d^, Carina Francisca Argüelles^d^, Marta Dolores Mudry^a, b^.*

**Supporting information captions**

**Table S1**

Positions of the 59 variable sites within the 515 bp fragment of the mtDNA-CR that define the 14 haplotypes in *Sapajus cay* and *Sapajus nigritus* individuals

Table S1. Positions of the 59 variable sites within the 515 bp fragment of the mtCR that define the 14 haplotypes in *Sapajus cay* and *Sapajus nigritus* individuals.

|  | 8 | | 21 | | 46 | | 48 | | 73 | | 75 | | 77 | | 80 | | 90 | | 93 | | 94 | | 95 | | 100 | | 102 | | 117 | | 125 | | 128 | | 129 | | 134 | | 144 | | 146 | | 150 | | 154 | | 161 | |
| --- | --- | --- | --- | --- | --- | --- | --- | --- | --- | --- | --- | --- | --- | --- | --- | --- | --- | --- | --- | --- | --- | --- | --- | --- | --- | --- | --- | --- | --- | --- | --- | --- | --- | --- | --- | --- | --- | --- | --- | --- | --- | --- | --- | --- | --- | --- | --- | --- |
| **H_1** | G | | A | | C | | C | | G | | A | | T | | A | | T | | C | | G | | C | | A | | A | | A | | A | | C | | C | | T | | A | | G | | T | | T | | C | |
| **H_2** | . | . | | . | | . | | . | | . | | . | | . | | . | | . | | . | | . | | . | | . | | . | | . | | . | | . | | . | | . | | . | | . | | . | | . | |  |
| **H_4** | . | . | | . | | . | | . | | . | | . | | . | | . | | . | | . | | . | | . | | . | | . | | . | | . | | . | | . | | . | | . | | . | | . | | . | |  |
| **H_7** | . | . | | . | | . | | . | | . | | . | | . | | . | | . | | . | | . | | . | | . | | . | | . | | . | | . | | . | | . | | . | | . | | . | | . | |  |
| **H_8** | . | T | | . | | . | | A | | G | | . | | . | | . | | . | | A | | . | | G | | G | | G | | . | | . | | . | | . | | . | | A | | C | | . | | T | |  |
| **H_9** | . | T | | . | | . | | A | | G | | . | | . | | . | | . | | A | | . | | . | | G | | G | | . | | . | | . | | . | | . | | A | | C | | . | | T | |  |
| **H_10** | A | T | | . | | . | | A | | G | | . | | . | | . | | . | | A | | . | | . | | G | | G | | . | | . | | . | | . | | . | | A | | C | | . | | T | |  |
| **H_13** | . | T | | . | | . | | A | | G | | . | | . | | . | | . | | A | | . | | . | | G | | G | | . | | . | | . | | . | | . | | A | | C | | . | | T | |  |
| **H_12** | . | T | | . | | . | | A | | G | | . | | . | | . | | . | | A | | . | | . | | G | | G | | . | | . | | . | | . | | . | | A | | C | | . | | T | |  |
| **H_5** | . | T | | . | | T | | . | | . | | . | | . | | . | | . | | . | | T | | . | | . | | . | | . | | . | | . | | . | | . | | A | | C | | . | | T | |  |
| **H_6** | . | T | | . | | . | | A | | . | | . | | . | | C | | . | | A | | . | | . | | . | | . | | G | | T | | T | | C | | . | | A | | . | | . | | T | |  |
| **H_14** | . | T | | . | | . | | A | | . | | . | | . | | C | | . | | A | | . | | . | | . | | . | | G | | T | | T | | C | | . | | A | | . | | . | | T | |  |
| **H_11** | . | T | | . | | . | | A | | . | | . | | . | | . | | . | | A | | . | | . | | . | | . | | G | | T | | T | | . | | . | | A | | . | | . | | T | |  |
| **H_3** | . | T | | T | | . | | . | | G | | T | | G | | . | | T | | A | | . | | G | | . | | G | | G | | T | | T | | . | | G | | . | | . | | C | | . | |  |

Dots indicate match to the most frequent haplotype, H_1

Table S1. Positions of the 59 variable sites within the 515 bp fragment of the mtCR that define the 14 haplotypes in *Sapajus cay* and *Sapajus nigritus* individuals (continued).

|  | 170 | 172 | | 174 | | 175 | | 176 | | 177 | | 178 | | 179 | | 185 | | 188 | | 190 | | 192 | | 193 | | 195 | | 196 | | 199 | | 213 | | 215 | | 224 | | 226 | | 231 | | 232 | | 233 | |
| --- | --- | --- | --- | --- | --- | --- | --- | --- | --- | --- | --- | --- | --- | --- | --- | --- | --- | --- | --- | --- | --- | --- | --- | --- | --- | --- | --- | --- | --- | --- | --- | --- | --- | --- | --- | --- | --- | --- | --- | --- | --- | --- | --- | --- | --- |
| **H_1** | C | A | | T | | A | | T | | A | | G | | T | | T | | A | | A | | T | | G | | C | | C | | T | | G | | T | | T | | C | | C | | A | | A | |
| **H_2** | . | . | | . | | . | | . | | . | | . | | . | | . | | . | | . | | C | | . | | . | | . | | . | | . | | . | | . | | . | | . | | . | | . | |
| **H_4** | . | . | | . | | . | | . | | . | | . | | . | . | | . | | . | | . | | . | | . | | . | | . | | . | | . | | . | | . | | T | | . | | . | |  |
| **H_7** | . | . | | . | | . | | . | | . | | . | | . | . | | . | | . | | . | | . | | . | | . | | . | | . | | . | | . | | . | | . | | . | | . | |  |
| **H_8** | . | . | | . | | . | | . | | . | | . | | . | . | | . | | G | | . | | . | | . | | . | | . | | A | | . | | . | | T | | T | | G | | . | |  |
| **H_9** | . | . | | . | | . | | . | | . | | . | | . | . | | . | | . | | . | | . | | . | | . | | . | | A | | . | | . | | T | | T | | G | | . | |  |
| **H_10** | . | . | | . | | . | | . | | . | | . | | . | . | | . | | . | | . | | . | | . | | . | | . | | A | | . | | . | | T | | T | | G | | . | |  |
| **H_13** | . | . | . | | . | | . | | . | | A | | . | | . | | . | | . | | . | | . | | . | | . | | . | | A | | . | | . | | T | | T | | G | | . | |  |
| **H_12** | . | . | . | | . | | . | | . | | . | | . | | . | | G | | . | | . | | . | | . | | . | | . | | A | | . | | . | | T | | . | | G | | . | |  |
| **H_5** | . | G | . | | . | | . | | G | | A | | C | | . | | . | | . | | . | | . | | . | | . | | . | | A | | .C | | . | | T | | . | | . | | G | |  |
| **H_6** | . | . | C | | . | | C | | G | | . | | . | | . | | . | | . | | . | | . | | T | | . | | .C | | A | | . | | . | | T | | . | | G | | . | |  |
| **H_14** | . | . | C | | . | | . | | G | | . | | . | | . | | . | | . | | . | | . | | T | | . | | C | | A | | . | | . | | T | | . | | G | | . | |  |
| **H_11** | . | . | C | | . | | C | | G | | . | | . | | . | | . | | . | | . | | . | | T | | . | | . | | A | | . | | C | | T | | . | | G | | . | |  |
| **H_3** | T | . | C | | G | | C | | G | | C | | . | | C | | . | | . | | . | | A | | . | | T | | . | | . | | . | | . | | . | | T | | . | | G | |  |

Dots indicate match to the most frequent haplotype, H_1

Table S1. Positions of the 59 variable sites within the 515 bp fragment of the mtCR that define the 14 haplotypes in *Sapajus cay* and *Sapajus nigritus* individuals (continued).

|  | 234 | 238 | 253 | 257 | 261 | 293 | 294 | 297 | 408 | 445 | 447 | 463 | G.A.N*. |
| --- | --- | --- | --- | --- | --- | --- | --- | --- | --- | --- | --- | --- | --- |
| **H_1** | A | T | A | T | C | T | T | G. | T | G | G | C | KJ737380 |
| **H_2** | . | . | . | . | . | . | . | . | . | . | . | . | KJ737384 |
| **H_4** | . | . | . | . | . | . | . | . | . | . | . | . | KJ737395 |
| **H_7** | . | . | . | . | . | . | C | . | . | . | . | . | KJ737406 |
| **H_8** | . | C | G | . | . | . | C | A | . | . | . | T | KJ737407 |
| **H_9** | . | C | G | . | . | . | C | A | . | . | . | T | KJ737416 |
| **H_10** | . | C | G | . | . | . | C | . | . | . | . | T | KJ737410 |
| **H_13** | . | C | G | . | . | . | C | A | . | . | . | T | KJ737414 |
| **H_12** | . | C | G | . | . | . | C | A | . | . | . | T | KJ737413 |
| **H_5** | . | . | . | . | . | . | C | A | . | . | . | T | KJ737404 |
| **H_6** | . | . | . | . | . | . | C | A | . | . | . | T | KJ737405 |
| **H_14** | . | . | . | . | . | . | C | A | . | . | . | T | KJ737415 |
| **H_11** | . | . | . | . | . | . | C | A | . | . | . | T | KJ737412 |
| **H_3** | G | . | G | C | T | C | C | . | C | A | A | T | KJ737388 |

Dots indicate match to the most frequent haplotype, H_1. * GenBank Accession Number
